# Forward and backward locomotion patterns in *C. elegans* generated by a connectome-based model simulation

**DOI:** 10.1038/s41598-021-92690-2

**Published:** 2021-07-02

**Authors:** Kazuma Sakamoto, Zu Soh, Michiyo Suzuki, Yuichi Iino, Toshio Tsuji

**Affiliations:** 1 Department of System Cybernetics, Graduate School of Engineering, Hiroshima University, Higashi-Hiroshima, Hiroshima, Japan; 2 Graduate School of Advanced Science and Engineering, Hiroshima University, Higashi-Hiroshima, Hiroshima, Japan; 3 Department of Radiation-Applied Biology Research, National Institutes for Quantum and Radiological Science and Technology, Takasaki, Gunma Japan; 4 Department of Biological Sciences, Graduate School of Science, The University of Tokyo, Bunkyo-ku, Tokyo, Japan

**Keywords:** Computational biology and bioinformatics, Neuroscience

## Abstract

*Caenorhabditis elegans* (*C. elegans*) can produce various motion patterns despite having only 69 motor neurons and 95 muscle cells. Previous studies successfully elucidate the connectome and role of the respective motor neuron classes related to movement. However, these models have not analyzed the distribution of the synaptic and gap connection weights. In this study, we examined whether a motor neuron and muscle network can generate oscillations for both forward and backward movement and analyzed the distribution of the trained synaptic and gap connection weights through a machine learning approach. This paper presents a connectome-based neural network model consisting of motor neurons of classes A, B, D, AS, and muscle, considering both synaptic and gap connections. A supervised learning method called backpropagation through time was adapted to train the connection parameters by feeding teacher data composed of the command neuron input and muscle cell activation. Simulation results confirmed that the motor neuron circuit could generate oscillations with different phase patterns corresponding to forward and backward movement, and could be switched at arbitrary times according to the binary inputs simulating the output of command neurons. Subsequently, we confirmed that the trained synaptic and gap connection weights followed a Boltzmann-type distribution. It should be noted that the proposed model can be trained to reproduce the activity patterns measured for an animal (HRB4 strain). Therefore, the supervised learning approach adopted in this study may allow further analysis of complex activity patterns associated with movements.

## Introduction

*Caenorhabditis elegans* (*C. elegans*), measuring approximately 1.0 mm in length and weighing 0.5 µg, is one of the most commonly studied multicellular organisms. Its body consists of approximately 1000 cells, including 302 neurons composing the nervous system^2^. Despite this low number of neurons, the worm produces various movement patterns by adjusting its whole-body movement according to diverse environmental stimuli^1–4^. Thus, the worm is a suitable model animal for investigating the mechanism of motion pattern generation.

**Figure 1:**
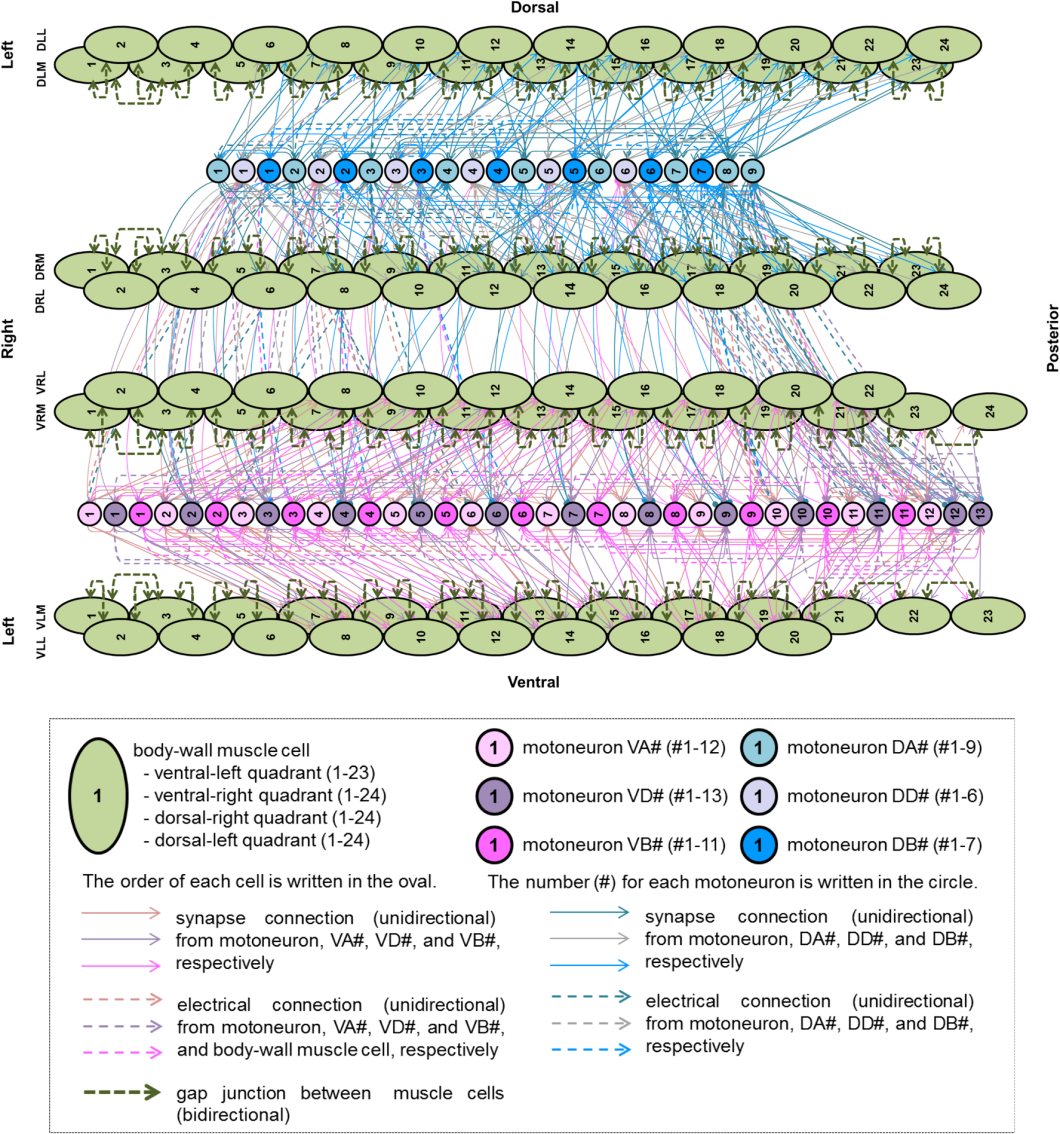
Diagram of neuro-muscular connections illustrated based on the WormAtlas database^6^. Synaptic connections and gap junctions of whole muscle cells (total 95) and motor neurons VA, VD, VB, DA, DD, and DB. The green oval represents the body-wall muscles, the left side of the figure is the anterior direction, the right side is the posterior direction, and, starting from the upper row, it shows the left dorsal side, right dorsal side, right ventral side, and left ventral side. The circles represent motor neurons, and the difference in color corresponds to the types of motor neurons, as shown in the legend. Solid lines represent synaptic connections, and dotted lines represent gap junctions.

The neural circuit structure related to motion generation has been clarified to some extent by biological experiments. Chalfie et al. revealed the role of interneurons that control forward and backward movement through experiments involving laser irradiation^1^. The anatomical study carried out by White et al. successfully mapped the connectome of *C. elegans*^5^, and the updated data can be accessed through the web database WormAtlas^6^. Figure 1 shows the neuromuscular connectome related to movement based on the connectome database (accessible at https://wormwiring.org/series/). Further experimental studies revealed the functions of the neural circuit structure. For example, Wen et al.^7^ found that proprioceptive feedback is essential for motion generation, and Kawano et al.^8^ showed that gap junctions are important for motion generation.

Although the structure and role of neurons related to movement have been clarified by experimental methods, explicating the function of the neural circuit requires the measurement and interpretation of the activity of each neuron. However, performing these tasks using only a bottom-up experimental approach would be extremely difficult. Therefore, a top-down approach using a mathematical model has been employed. Niebur and Erdös constructed a body dynamics model incorporating the central pattern generator to analyze the forward movement of *C. elegans*^9^, and postulated that stretch receptors are important for the generation of movement. Bryden and Cohen analyzed the gait generation mechanism of forward movement using a body model incorporating the command neuron AVB and motor neurons^10^. An integrated neuromechanical model proposed by Cohen’s group^10–12^ is often used in recent research to study neuromuscular control. Olivares et al.^13^ presented a model that incorporates the connectome and verified the existence of both a central pattern generator in the ventral nerve cord and the role of command neurons in switching between forward and backward motion. Fieseler et al.^14^ found that suppression of proprioceptive feedback may contribute to the generation of an omega turn. Although various aspects of motion generation in *C. elegans* have been elucidated, these models did not analyze the distribution of the synaptic and gap connection strength. According to observation in the actual animal, it was found that the number of synapses follows a modified Boltzmann distribution^15^. This modified Boltzmann distribution indicates that the network is sparsely connected.

This study examines whether the motor neuron and muscle network could generate oscillation patterns for both forward and backward movement, and analyzes the distribution of the synapse and gap connection weights. The proposed model consists of motor neurons of classes A, B, D, AS, and muscle. Both gap junctions and synaptic connections are included based on the connectome data. The input to the motor neurons from the command neurons is modeled as a binary input based on their role in evoking forward and backward movements^1,8^, in addition to the generated teacher data from measured muscle activities of a strain expressing calcium indicator in the body-wall muscle (HRB4). The connection parameters were then trained using a supervised algorithm. Finally, the trained model was driven by the untrained input of the command neuron to examine its generalizability, and the fit of trained weights to the modified Boltzmann distribution was tested.

## Materials and methods

### Assumptions

Figure 1 shows the connection diagram of the model based on the connectome described in the nematode database WormAtlas^6^. In particular, the model consists of seven types of 69 motor neurons that receive five pairs of ten binary inputs, and four rows of 95 muscles^5,6,16^. The neurons and muscles are connected based on the adjacency matrix of the synaptic and gap connections accessible from the following https://wormwiring.org/pages/adjacency.html^6^.

We considered the inputs to motor neurons from command neurons of PVCL/R and AVBL/R (responsible for forward movement), in addition to AVAL/R, AVDL/R, and AVEL/R (responsible for backward movement)^1^. We assumed that these inputs have no specific polarity, and do not include an oscillating component to generate periodic muscle contractions^17–20^.

Motor neurons are comprised of DB, DA, DD, VB, VD, VA, and AS (indicated by circles in Fig. 1). Cholinergic neurons, DB, DA, VB, VA, and AS were assumed to excite VD, DD, and muscles. GABAergic neurons VD and DD then inhibit the cholinergic neurons and muscles. Although the polarities of the synaptic connections were determined based on the description in a previous study^5,6^, they have not been fully confirmed experimentally. Therefore, it should be noted that these connections have the potential to be either excitatory or inhibitory in an actual animal.

Proprioceptive feedback also plays an important role in generating undulatory movements, and the undifferentiated processes extending from A- and B-class motor neurons are responsible for proprioceptive feedback^7^. Because the model does not contain the body in order to focus on the neuromuscular system, we connected several muscles to an anterior A-class neuron and a posterior B-class neuron to represent proprioceptive feedback, assuming that muscle activity correlates with body curvature. Here, we did not impose the polarities of the proprioceptive feedback connections.

In Fig. 1, the *C. elegans* muscle indicated by the ellipse is composed of 95 cells arranged in four rows, and the ventral and dorsal muscle cells alternately contract and relax to produce a smooth crawling motion. For simplification, we assumed the simultaneous activation in each left and right muscle pair, considering their two-dimensional movement on the agar. We trained the model using backpropagation through time (BPTT), a supervised algorithm, to generate muscle activity patterns using two types of teacher data: a mathematically defined sinusoidal pattern, and an activity pattern measured from a fluorescence strain (HBR4).

### Mathematical expression of the model

The model considers inputs from forward command neurons (PVCL/R, AVBL/R) and backward command neurons (AVAL/R, AVBL/R, AVEL/R), which are modeled to output control signals of 1 or 0. An output of 1 represents the activated state, and 0 represents the resting state. As shown in the following equations, the forward command neuron $L_{a} \left( t \right)$ outputs 1 and the backward command neuron $L_{c} \left( t \right)$ outputs 0 during a preset time $[T_{{2v - 1}} ,T_{{2v}} )~\left( {v = 1,~2, \ldots ,~o} \right)$ to command forward movement. This output is reversed during $\left[ {T_{{2v}} ,T_{{2v + 1}} } \right)$ to command backward movement:

1 $$L_{a} \left( t \right) = \left\{ {\begin{array}{*{20}c} 1 & {\left( {T_{{2v - 1}} \le t < T_{{2v}} } \right)} \\ 0 & {\left( {T_{{2v}} \le t < T_{{2v + 1}} } \right)} \\ \end{array} } \right.,$$

2 $$L_{c} \left( t \right) = \left\{ {\begin{array}{*{20}c} 0 & {\left( {T_{{2v - 1}} \le t < T_{{2v}} } \right)} \\ 1 & {\left( {T_{{2v}} \le t < T_{{2v + 1}} } \right)} \\ \end{array} } \right.,$$

where ${\text{~}}L_{a} \left( t \right)$ is the output of the forward command neurons (PVCL/R, AVBL/R) and $L_{c} \left( t \right)$ is the output of the backward command neurons (AVAL/R, AVBL/R, AVEL/R). The index *a* = 1, 2, 3, 4 corresponds to a total of four forward command neurons, and *c* = 5, 6, …, 10 corresponds to a total of six backward command neurons.

In the motor neurons, a muscle contraction pattern is generated based on the binary input from the command neurons. The motor neuron is defined by the following equations:

3 $$\begin{aligned} x_{i}^{N} \left( {t + 1} \right) &= \frac{1}{{1 + F_{s} \tau _{i} }}x_{i}^{N} \left( t \right) \\ & \quad + \frac{{F_{s} \tau _{i} }}{{1 + F_{s} \tau _{i} }}\left\{ {\sum\limits_{{j = 1}}^{J} {w_{{ij}}^{{NN}} y_{j}^{N} (t) + } \sum\limits_{{u = 1}}^{U} {w_{{iu}}^{{MN}} y_{u}^{M} (t) + \sum\limits_{{j = 1}}^{J} {g_{{ij}}^{{NN}} \left( {x_{j}^{N} (t) - x_{i}^{N} (t)} \right) + } \sum\limits_{{u = 1}}^{U} {g_{{iu}}^{{MN}} \left( {x_{u}^{M} (t) - x_{i}^{N} (t)} \right) + } \sum\limits_{{p = 1}}^{p} {w_{{ip}}^{{IN}} L_{p} (t) + I_{{{\text{p}}i}} (t) + w_{i}^{{l0}} } } } \right\} \\ \end{aligned}$$

4 $$y_{i}^{N} \left( t \right) = \frac{1}{{1 + \exp \left( { - x_{i}^{N} \left( t \right)} \right)}}$$

where $x_{i}^{N} \left( t \right)$ and $x_{u}^{M} \left( t \right)$ are currents flowing into motor neuron *i* and muscle *u* at time *t,* respectively; $y_{i}^{N} \left( t \right)$ and $y_{u}^{M} \left( t \right)$ are membrane potentials of motor neurons and muscles, respectively; $\omega _{i}^{{l0}}$ is the bias, $\tau _{i}$ is the first-order lag element, and *F*_*s*_ is the sampling frequency configured in the simulation procedure. $\omega _{{ij}}^{{NN}}$, $\omega _{{iu}}^{{MN}}$, $\omega _{{ip}}^{{IN}}$ are weights representing the synaptic connection strength among motor neurons, that from muscle to motor neuron, and that from the command neuron to motor neuron, respectively.$~g_{{ij}}^{{NN}} = g_{{ji}}^{{NN}} ,~~g_{{iu}}^{{MN}} = g_{{ui}}^{{MN}}$ are weights corresponding to the conductance of the gap junctions. *J* = 69 is the number of motor neurons, *P* = 10 is the number of command neurons, and *U* = 95 is the number of muscle cells. $I_{{{\text{p}},i}} \left( t \right)$ represents the proprioceptive feedback to the A- and B-class motor neurons determined by the following equation.

5 $$I_{{{\text{p}}i}} \left( t \right) = \left\{ {\begin{array}{*{20}c} {\mathop \sum \limits_{{u = 1}}^{S} w_{{i\left\{ {3\left( {i + 1} \right) - u} \right\}}}^{{MN}} y_{{\left\{ {3\left( {i + 1} \right) - u} \right\}}}^{M} \left( t \right),} & {i \in {\text{B}} - {\text{class~}}\;{\text{motor}}\;{\text{~neuron}}} \\ {\mathop \sum \limits_{{u = 1}}^{S} w_{{i\left\{ {3\left( {i + 1} \right) + u} \right\}}}^{{MN}} y_{{\left\{ {3\left( {i + 1} \right) + u} \right\}}}^{M} \left( t \right),} & {i \in {\text{A}} - {\text{class~}}\;{\text{motor}}\;{\text{~neuron}}} \\ {0,} & {{\text{Otherwise}}} \\ \end{array} } \right.$$

Here, $~w_{{i\left\{ {3\left( {i + 1} \right) - u} \right\}}}^{{MN}}$ denotes the connection strength, $3\left( {i + 1} \right) - u$ and $3\left( {i + 1} \right) + u$ are the indexes of a muscle, and we set the proprioceptive feedback length as $S = 7$ muscles. For the motor neurons located at the head and tail, *S* was set to the number of the existing anterior and posterior muscles. In this configuration, an A- and B-class neuron receives the outputs from up to seven anterior and posterior muscles.

When the muscles receive the output from the motor neurons, they output the muscle activation level according to the following equations:

6 $$\begin{aligned} & x_{u}^{M} \left( {t + 1} \right) = \frac{1}{{1 + F_{s} \tau _{u} }}x_{u}^{M} \left( t \right) \\ & \quad + \frac{{F_{s} \tau _{u} }}{{1 + F_{s} \tau _{u} }}\left\{ {\mathop \sum \limits_{{j = 1}}^{J} \omega _{{uj}}^{{NM}} y_{j}^{N} \left( t \right) + \mathop \sum \limits_{{j = 1}}^{J} g_{{uj}}^{{MN}} \left( {x_{j}^{N} \left( t \right) - x_{u}^{M} \left( t \right)} \right) + \mathop \sum \limits_{{k = 1}}^{K} g_{{uk}}^{{MM}} \left( {x_{k}^{M} \left( t \right) - x_{u}^{M} \left( t \right)} \right) + \omega _{u}^{{l1}} } \right\} \\ \end{aligned}$$

7 $$y_{u}^{M} \left( t \right) = \frac{1}{{1 + \exp \left( { - x_{u}^{M} \left( t \right)} \right)}},$$

where $\omega _{{uj}}^{{NM}}$ is a weight representing the synaptic connection strength from the motor neuron to muscle, and $g_{{uk}}^{{MM}}$ is a weight representing the conductance of the gap junctions between the muscle, *K* = 95 is the number of muscle cells.

According to the connectome data^6^, the absence of connections was represented by restricting the weights of the synapse connection and gap junction to 0 during the following training process.

### Training algorithm

The parameters of the neural network model (first-order lag element, synaptic connections, and gap junctions) are trained using the BPTT algorithm^21^. The evaluation function is defined as:

8 $$E = \frac{1}{T}\frac{1}{U}\mathop \sum \limits_{{t = 1}}^{T} \mathop \sum \limits_{u}^{U} \frac{1}{2}\left\{ {y_{u}^{M} \left( t \right) - d_{u} \left( t \right)} \right\}^{2} ,~~$$

where $d_{u} \left( t \right)$ is the teacher data, $y_{u}^{M} \left( t \right)$ is the activity level of the muscles, and *T* is the maximum step number of the simulation time. BPTT updates each parameter in a particular direction to minimize the evaluation function, which is determined by partial differentiation of the evaluation function for the corresponding parameter. In addition, the following constraint condition is set for each parameter based on the type of connections.

・The synaptic connection strengths from the excitatory neurons DA ($i = 1,~2, \ldots ,~8$), DB ($i = 9,~10, \ldots ,~16$), VA ($i = 23,~24, \ldots ,~34$), VB ($i = 35,~36,~ \ldots ,~45$), and AS ($i = 59,~60,~ \ldots ,~69$) are constrained to positive values.

The synaptic connection strengths from inhibitory neurons DD ($i = 17,~18,~~ \ldots ,~22$) and VD ($i = 46,~47,~~ \ldots ,~58$) are constrained to negative values.

・The first-order lag elements ${\text{~}}\tau _{i}$,${\text{~}}\tau _{u}$ are constrained to positive values ($\tau _{i} \ge 0$).

**Figure 2:**
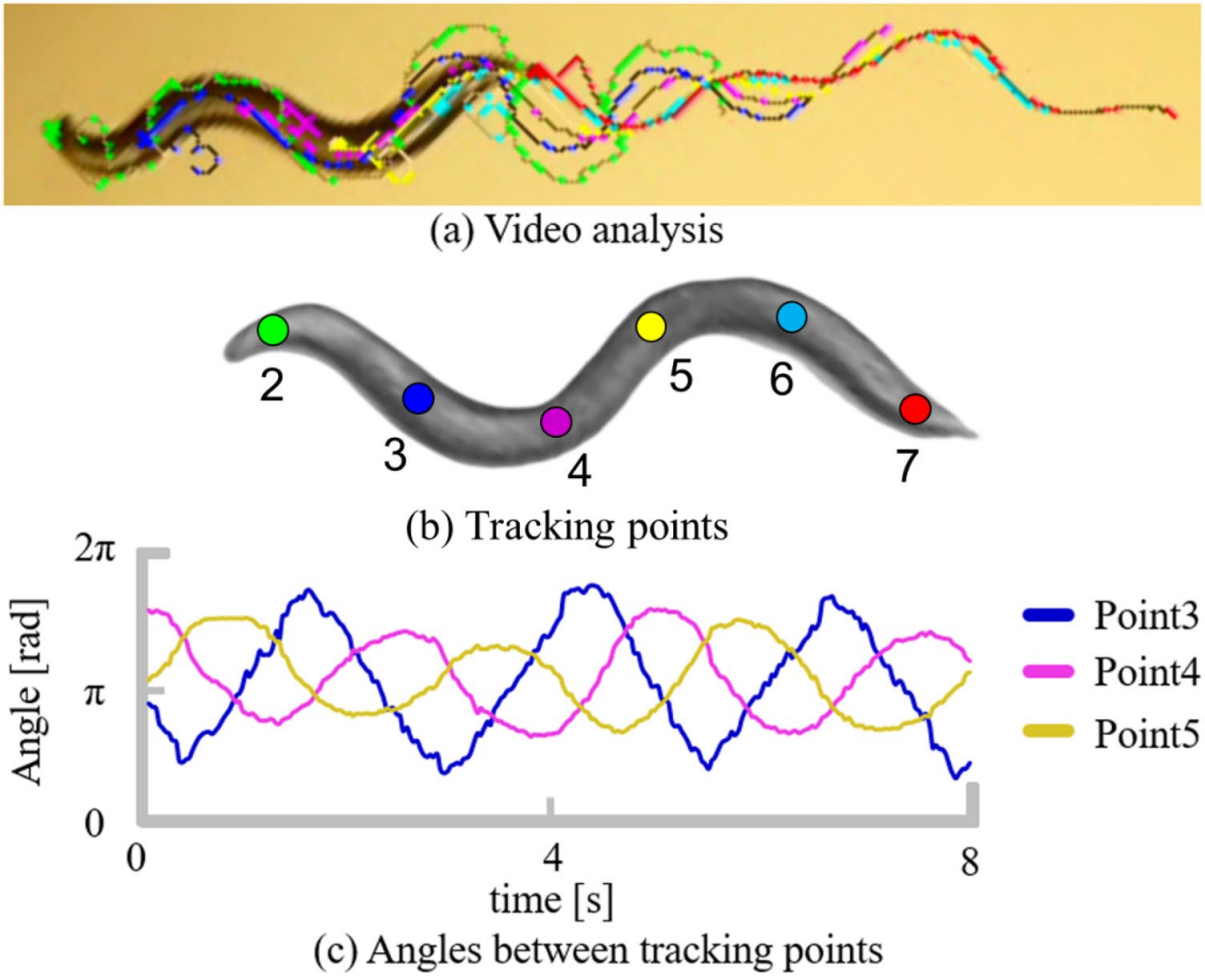
Motion analysis of *C. elegans.* (**A**) Motion analysis capture of *C. elegans* using the image analysis software Wriggle tracker (Library Inc., Tokyo). (**B**) Points tracked by the image analysis software. (**C**) Temporal change in the angles between the straight lines connecting adjacent points.

The teacher data $d_{u} \left( t \right)$ used for parameter adjustment are generated based on the motion analysis of *C. elegans*. As shown in Fig. 2, we tracked seven points on the body of *C. elegans* while performing forward movement and plotted the time change of the angles between the two straight lines connecting adjacent points. In the figure, each angle shows a sinusoidal-like waveform that is transmitted from the head to the tail. Because the movement of *C. elegans* is generated by alternating activities of the muscles, the activity level is considered to have similar characteristics. Therefore, we express the teacher data for the activity level of the muscles according to Eqs. (9)–(11).

In the first period of forward movement $\left( {T_{1} \le t < T_{2} } \right)$, when the index *q* = 1, 2, …, 24 is sequentially defined for the muscles from the head to the tail, the teacher data for the left dorsal (*u* = 1, 2, …, 24) and the right dorsal (*u* = 25, 26, …, 48) muscles are given by the following equation:

9 $$d_{u} \left( t \right) = \sin \left( {\omega t + \phi _{u} \left( t \right)} \right)\quad ~\left( {u = 1,~2, \ldots ,~24,~25,~26,~ \ldots ,~48} \right),~$$

where phase $\phi _{u} \left( t \right),~\left( {\phi _{u} \left( 0 \right) = - \frac{{\pi q}}{{12}}} \right)$ is changed when switching between forward and backward movement at a time $T_{v} \left( {v > 1} \right)$ as defined below.

10 $$\phi _{u} \left( t \right) = \pi - 2\omega T_{v} - \phi _{u} \left( {T_{{v - 1}} } \right),$$

Similarly, the left and right ventral muscle $\left( {u = 49,~50,~~ \ldots ,~72,~73,~74, \ldots ,~95} \right)$ are assumed to be active in opposite phases to the dorsal muscle cells, and are given by the following equation:

11 $$d_{u} \left( t \right) = \sin \left( {\omega t - \pi + \phi _{u} \left( t \right)} \right)\quad \left( {u = 49,~50,~~ \ldots ,~72,~73,~74, \ldots ,~95} \right).$$

Based on measured data, the angular frequency is set to *ω* = 1.6 $\pi$.

**Figure 3:**
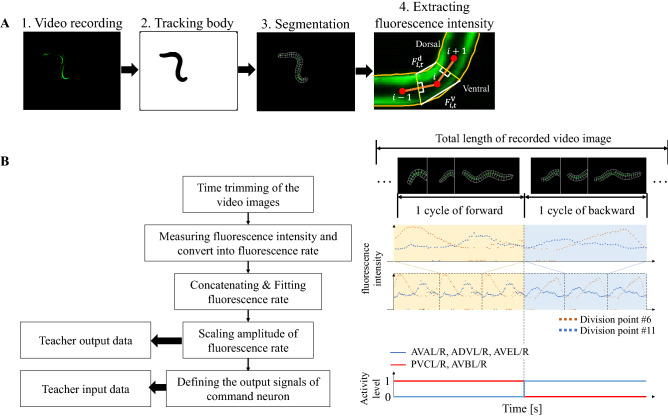
Measurement of fluorescence intensity. (**A**) The measurement procedure. The body is divided into *i* = 1, 2 …, 24 segments using a video analysis software (Worm-Lab, MBF Bioscience, Williston, USA). The average fluorescence intensities were extracted at the dorsal and ventral sides of the *i*-th segment. (**B**) Procedure of teacher data preparation. The left shows the block diagram of data preparation, and the right shows the corresponding images.

**Figure 4:**
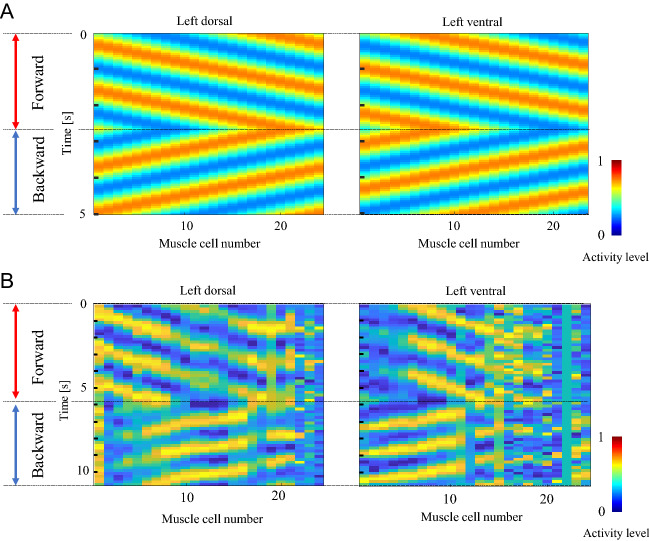
Teacher data for muscles. (**A**) Teacher data generated by Eqs. (9)–(11). (**B**) Teacher data generated using the measured muscle activity. The red arrows represent the time duration of forward movement, and the blue arrows represent the time duration of backward movement. The output switches between forward and backward movement at the predetermined time. The x-axis represents the muscle cell number, indexed from head to tail, the y-axis represents time, and the color represents the teacher data for the muscle activation level. The same teacher data are used for the right side of the body-wall muscles.

Figure 3 shows the procedure of preparing the second teacher dataset. For $d_{u} \left( t \right)$ of the second teacher dataset, the muscle activities were measured using a strain expressing calcium indicator in the body-wall muscle (HBR4: *goeIs3*[*pmyo-3*::*GCamP3*.*35*::*unc-54-3*' *utr*, *unc-119*(+)]*V*), and the model was trained using the measured fluorescence rate. The specification of the fluorescence microscope system was same as that described in the literature^22^ and the locomotion was video-recorded based on procedures^3^ previously established for wild-type animals. The recorded video images were then trimmed to extract the duration of one cycle of forward movement, followed by one cycle of backward movement. As shown in Fig. 3, a video analysis software (WormLab, MBF Bioscience, Williston, USA) was used to track the body outline of the animal, and then the body was divided into 24 equal segments from the head to the tail. The fluorescence intensities at the dorsal (*k* = d) and ventral (*k* = v) sides of the divided *i*-th segment $F_{{i,t}}^{k}$ were measured in the ventral and dorsal areas of each segment, and converted to fluorescence rates $\left( {R_{{i,t}}^{k} = \left( {F_{{i,t}}^{k} - F_{{0,i}}^{k} } \right)/F_{{0,i}}^{k} } \right),$ where $F_{{0,i}}^{k}$ is the minimum fluorescence intensity at the *k* side of the *i*-th segment. $R_{{i,t}}^{k}$ was concatenated to generate several cycles of forward and backward movement as shown in Fig. 4, and fitted to multiple sinusoidal functions, as expressed by the following equation, to smooth the measured data:

12 $$d_{u} \left( t \right) = \mathop \sum \limits_{{i = 1}}^{n} a_{{ui}} {\text{sin}}\left( {\omega _{{ui}} t + \phi _{{ui}} } \right),$$

where $n = 8$ is the number of the functions,* t* is the time, $a_{{ui}}$, $\omega _{{ui}}$, ${\text{and~}}\phi _{{ui}}$ are the amplitude, frequency, and phase of the *i*-th sinusoidal function, respectively. The fitted data were then concatenated to generate three cycles of forward and backward movement. The value of fluorescence intensity was normalized to a range [0.25, 0.75], and used as the teacher data to train the model.

## Simulation conditions

The teacher data for training was configured as follows: the simulation time was set to *T* = 30 s, and the sampling frequency was set to *F*_*s*_ = 0.05 s, the time for switching between forward and backward movement was set to *T*_1_ = 0.0, *T*_2_ = 8.7, *T*_3_ = 17.6, *T*_4_ = 22.8, *T*_5_ = 26.6. The initial value of each parameter was set at random according to a uniform distribution within the following range.

The excitatory synaptic connection $\omega _{{ij}}^{{NN}} ,{\text{~}}\left( {i = 1{-}8,{\text{~~}}9{-}16,{\text{~~}}23{-}34,{\text{~~}}35{-}45,{\text{~}}{-}69} \right)$: [0, 1]

$$\omega _{{ui}}^{{NM}} ,~\left( {j = 1{-}8,{\text{~~}}9{-}16,{\text{~~}}23{-}34,{\text{~~}}35{-}45} \right):{\text{~}}\left[ {0,{\text{~}}1} \right]~$$

The inhibitory synaptic connection $\omega _{{ij}}^{{NN}} ,{\text{~~}}\left( {i = 17{-}22,{\text{~~}}46{-}58} \right)$: [− 1, 0]

$$\omega _{{ui}}^{{NM}} ,~\left( {i = 17{-}22,{\text{~~}}46{-}58} \right):{\text{ }}\left[ { - {\text{1}},{\text{ }}0} \right]$$

The synaptic connections $\omega _{{iu}}^{{MN}} ,{\text{~~}}\omega _{{ip}}^{{IN}} ,{\text{~~}}\omega _{i}^{{l0}} ,~~\omega _{u}^{{l1}}$: [− 1, 1].

The gap junctions $g_{{ij}}^{{NN}} ,~~g_{{iu}}^{{MN}} ,{\text{~~}}g_{{uk}}^{{MM}}$: [0, 1].

The first-order lag elements $\tau _{i} ,~~\tau _{u}$: [0, 0.01].

The iterative adjustment of parameters terminates when the evaluation function (Eq. (8)) satisfies *E* ≤ 0.005.

Figure 4 A illustrates the teacher data generated using Eqs. (1), (2), and (9), and B shows the teacher data generated using the measured muscle activity. The same teacher data were used to train the ipsilateral side, assuming movement on the 2D agar plane. The left part of Fig. 4 shows the binary input from the command neurons. Color maps show the teacher data of the muscles. This figure shows that during forward movement, waves are transmitted from the head to the tail with some phase delay. Conversely, the waves are transmitted from the tail to the head during the period of backward movement. The phase of each wave is reversed at the time when the forward and backward movement switches.

## Results

### Training of model parameters

**Figure 5:**
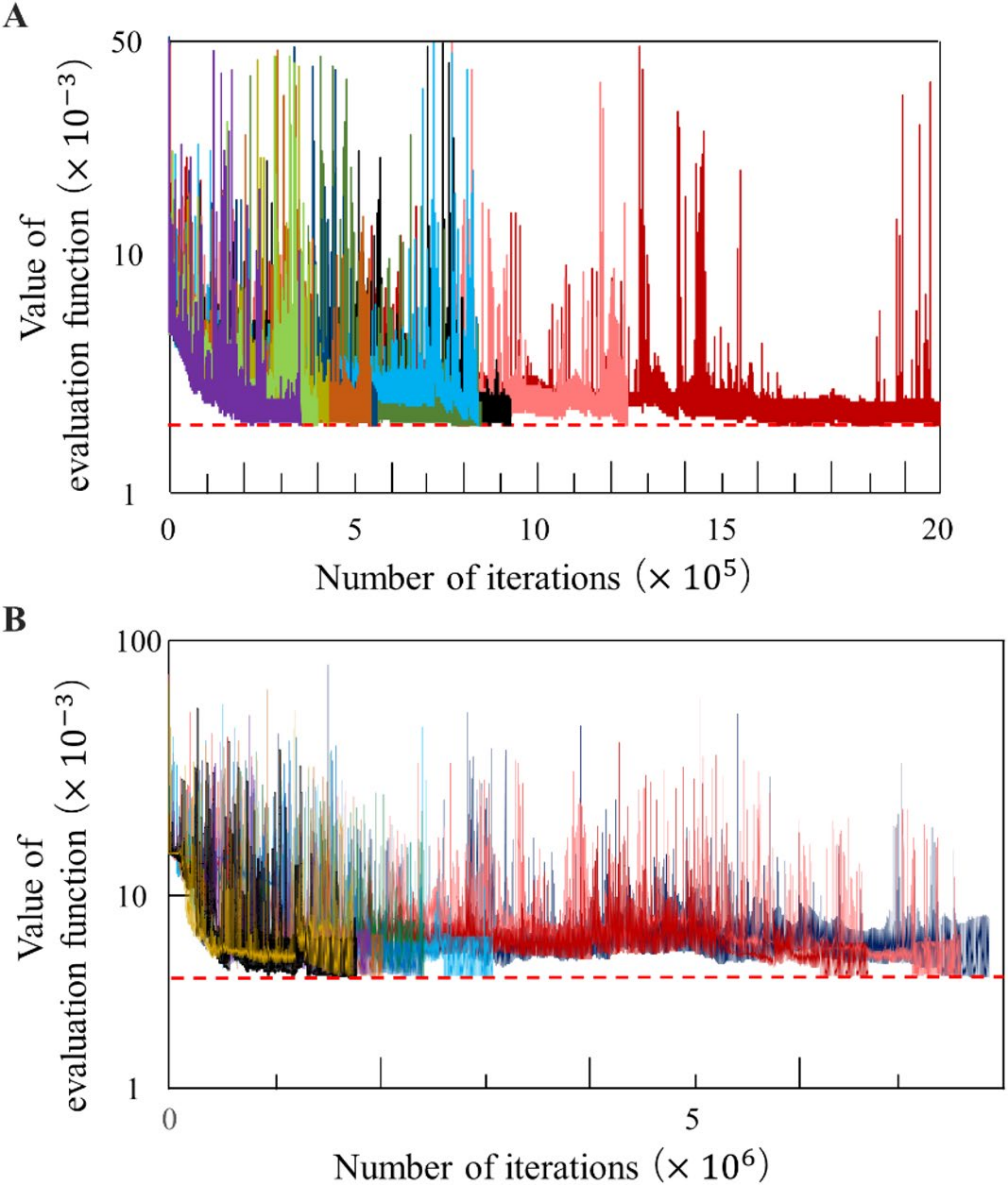
Learning curve. The values of evaluation function converged through the BPTT learning algorithm for all five trials. The dotted red line represents the error tolerance. (**A**) Learning curve obtained from ten training trials using the teacher data generated from Eqs. (9)–(11). (**B**) Learning curve obtained from ten training trials using the teacher data generated from the measured muscle activity.

Figure 5 illustrates the convergence of the residual errors defined by the evaluation function (Eq. (8)) over ten training trials using the teacher data generated from Eqs. (9)–(11) and another ten training trials using teacher data generated from the measured muscle activity. By adjusting the model parameters, the evaluation function decreased as the learning iterations increased, and finally reached an error tolerance of *E* ≤ 0.005, although the convergence speeds differ depending on the random initial parameters.

**Figure 6:**
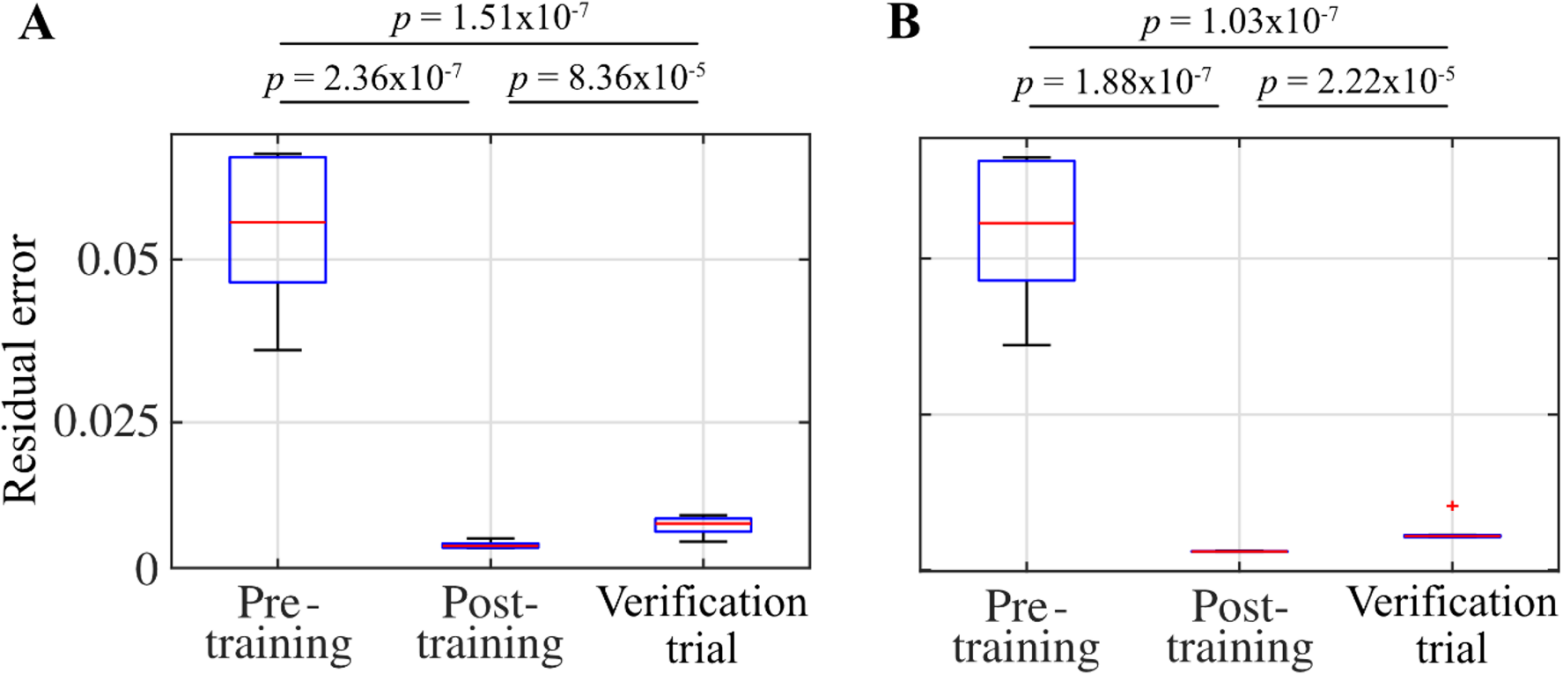
Residual errors. (**A**) and (**B**) show the residual errors (see Eq. (8)) of the models trained by artificial teacher signals and measured muscle activity, respectively. Multiple comparisons with Bonferroni adjustment illustrate significant differences between the residual errors of the pre-training and post-training, and pre-training and verification trials (*p* < 0.001).

Figure 6 shows the residual errors of pre-training, post-training, and verification trials. Significant differences (*p* < 0.001) between pre-training and post-training, and pre-training and verification trials indicate that the model was successfully trained. Although significant differences were also found between the post-training and verification trials, we confirmed that the model could generate the oscillation of muscle activity and switch between oscillation patterns for forward and backward movements, as described below.

### Muscle activity generated by the trained model

**Figure 7:**
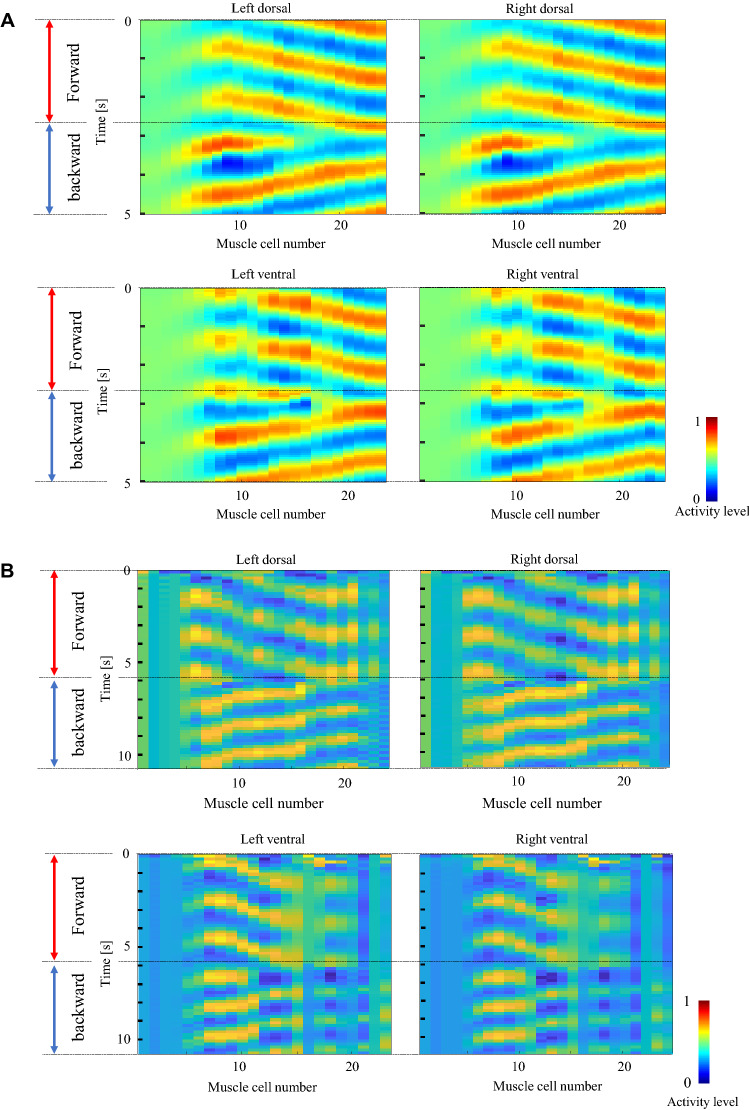
Muscle activities generated by the trained input. The phases of the muscle activity are reversed as the inputs of the command neurons change. The four muscles from the head in each quadrant, controlled by the nerve ring, fail to generate oscillation patterns. In (**A**), the model is trained by the teacher data generated from Eqs. (9)–(11). In (**B**), the model is trained by the teacher data generated from the measured muscle activity.

Figure 7 shows examples of muscle activities generated by feeding trained input to the model. The two sample data shown in Fig. 7 are those with the median values of residual errors among the ten trials of training, respectively, for the two types of teacher data. The figure confirms that the muscle activity patterns corresponding to forward and backward movement are successfully generated based on the inputs from the command neurons. The muscle activity propagates from head to tail when the forward command neurons activate, and it propagates from tail to head when the backward command neurons activate. However, the model failed to generate activities in neck muscles, as shown in the output of the four–eight muscles from the head in each quadrant. This is because the model focuses on the ventral cord motor neurons and does not include the nerve ring motor neurons. The nerve ring is a neuropile involved in processing various information related to various behaviors in addition to forward and backward movement^5^. Four muscles from the head in each quadrant are innervated by the nerve ring motor neurons, and the next four muscles each in the head are innervated by both the body-wall motor neurons and head motor neurons^23^. This result indicates that the gap junctions between the muscles cannot transmit the activity pattern to the four muscles in the head, and these muscle cells are independently controlled by the nerve ring motor neurons.

**Figure 8:**
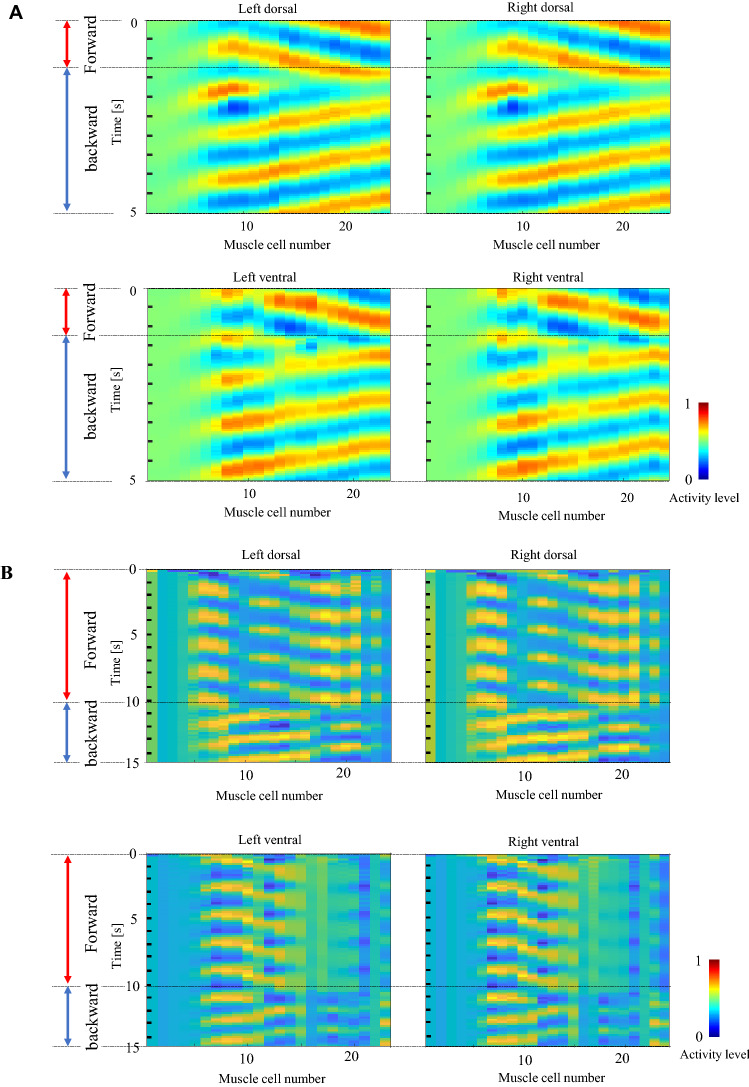
Muscle activities generated by the verification input. (**A**) and (**B**) show examples of the muscle activity generated by the trained models in a verification trial, which yielded the median value of the residual errors. The activity patterns corresponding to the forward and backward movements are successfully generated by switching the phase delay, as with the training data.

In the verification simulation, we inputted command neuron signals to evoke switching between forward and backward movement at different points in time from those in the teacher data. The switching times are *T*_1_ = 0.0, *T*_2_ = 4.7, *T*_3_ = 7.3, *T*_4_ = 17.25, *T*_5_ = 22.5, and *T*_6_ = 28 s for the model trained by the teacher data generated by Eqs. (9)–(11), and *T*_1_ = 0.0, *T*_2_ = 10.1 s for the model trained by the teacher data generated from the measured muscle activity. The outputs of the muscles are shown in Fig. 8. Again, the two sample data shown in Fig. 8 are those with the median values of residual errors among the ten training trials for the two types of teacher data. The results confirm that muscle activity patterns corresponding to forward and backward movement are successfully generated, as with the training data. This result indicates the generalizability of the time of switching between the forward and backward movements.

### Distribution of synaptic and conductance weights

**Figure 9:**
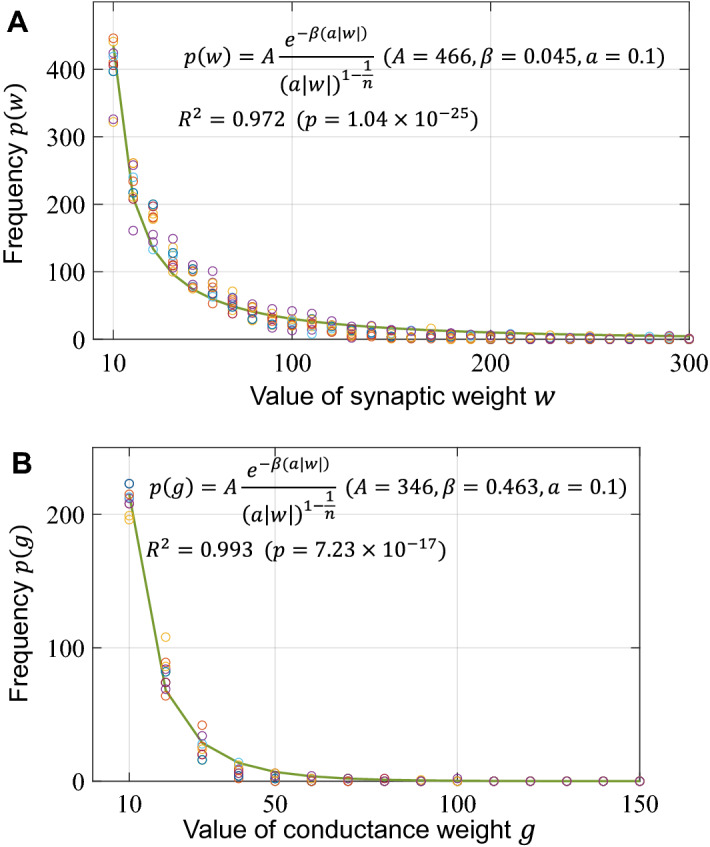
Distribution of weight parameters. (**A**) Frequencies in the corresponding bins as a function of the synaptic weights. (**B**) Frequencies as a function of the conductance weights. The solid green lines represent the fit to the modified Boltzmann distribution. The distributions were obtained from 10 training trials with different initial values. The colors of the circles in the plots distinguish the different training trials.

Finally, we analyzed the trained parameters, which are synaptic and conductance weights. An analysis by Snider^15^ showed that the number of synapses in the actual animal follows the modified Boltzmann distribution given by the following equation:

13 $$p\left( w \right) = A\frac{{e^{{ - \beta \left( {a\left| w \right|} \right)}} }}{{\left( {a\left| w \right|} \right)^{{1 - \frac{1}{n}}} }}~~~~,$$

where $A$ and $a$ are the scaling factors, $\beta$ is the power index, and $n$ is the total number of synaptic connections. We adopted this equation to fit the strength distribution of the synaptic and conductance weights. Figure 9 plots the distribution of the trained synaptic and conductance weights for each of the 10 training trials. The frequencies of weights in all training trials showed a similar trend of decreasing exponentially with the weight strength. Figure 9 also shows the fitted line of the modified Boltzmann distribution to the mean frequency. The distribution of the trained synaptic weights and conductance weights fitted the modified Boltzmann distribution well, with coefficients of determination $R^{2} = 0.972$ ($p = 1.04 \times 10^{{ - 25}}$) and $R^{2} = 0.993$ ($p = 7.23 \times 10^{{ - 17}}$), respectively. This result indicates the possibility that not only the number of synapses but also the synaptic strength could follow the modified Boltzmann distribution. In addition, Fig. 9(b) predicts that the conductance of the gap connection could also follow the modified Boltzmann distribution.

Theoretically, synapse connections following the modified Boltzmann distribution form a candidate network structures that can maximize the information storage^15^. In this study, the neural network model was trained to store the dynamic patterns corresponding to forward and backward motion using the BPTT algorithm, thus, as a consequence, the weights may have followed a modified Boltzmann distribution.

## Discussion

This study formulated a neural network model consisting of 69 motor neurons and 95 muscle cells based on the connectome of *C. elegans*. After the parameters of the model were adjusted using machine learning, it was shown that the motor neurons could generate the activity patterns of the muscle cells corresponding to forward and backward movement based on the input from the command neurons modeled as binary values. However, the four-eight muscles in the head failed to generate oscillation patterns. This result suggests that the gap junction between muscle cells cannot transmit activity to generate head motion, and the nerve ring motor neuron is necessary to control the activity of these muscles.

Previous models assigned the values of parameters by various means^11–14,24–28^, such as manually^10^ or by using an evolutional algorithm^13,26^. However, the distribution of the assigned parameter values was not analyzed. In this study, we trained the proposed model using the BPTT algorithm. We found that the modified Boltzmann distribution was well fitted to the distribution of the trained parameters (synaptic and conductance weights). This result predicts that the motor neuron and muscle network downstream of the command neurons could form a sparse network in terms of connection strength, which is advantageous to code various movement patterns^15^.

While the previous models reproduced the frequency and wavelength of muscle activities, the proposed model reproduced the fluorescence rates measured from the body wall muscles using a fluorescence strain (HBR4). Therefore, the supervised learning approach taken in this study may allow further analysis of complex activity patterns associated with movements because it provides a framework to reproduce the measured muscle activity patterns of an actual animal.

The proposed model does not consider body dynamics, which renders it unable to explore the ability of gait adaptation through proprioceptive feedback. However, these aspects of motion have already been extensively analyzed in previous models^11–14,24–28^. Incorporating body dynamics models^11,12,28–32^ is certainly required for the further analysis of complex movements such as an omega turn.

## Conclusion

In this paper, we presented a connectome-based neuromuscular network model of *C. elgans*. The model is trained to generate muscle oscillation patterns for both backward and forward movements using a supervised learning approach. The main finding of this study is that a motor neuron and muscle network with a sparse connection strength can generate the oscillatory patterns. In addition, the model can be trained to generate measured muscle activity patterns. Therefore, the supervised learning approach taken in this study may allow further analysis of complex activity patterns associated with movements.
